# Examination of Information Quality on Public Instagram Profiles Regarding Botulinum Toxin for Bruxism: A Study in São Paulo State, Brazil

**DOI:** 10.4317/jced.61767

**Published:** 2024-07-01

**Authors:** Alex-Moreira Mélo, Jéssica-Talaveiro Pacheco, Melissa-de Oliveira Melchior, Fabiane-Carneiro Lopes-Olhê, Jardel-Francisco Mazzi-Chaves, Laís-Valencise Magri

**Affiliations:** 1 DDS. Department of Restorative Dentistry, School of Dentistry of Ribeirão Preto, University of São Paulo, Ribeirão Preto, São Paulo - Brazil; 2 DDS. Course of Dentistry, University of Ribeirão Preto (UNAERP), Ribeirão Preto, São Paulo, Brazil; 3 MD. Department of Restorative Dentistry, School of Dentistry of Ribeirão Preto, University of São Paulo, Ribeirão Preto, São Paulo - Brazil; 4 DDS, MD, PhD. Department of Restorative Dentistry, School of Dentistry of Ribeirão Preto, University of São Paulo, Ribeirão Preto, São Paulo - Brazil

## Abstract

**Background:**

Currently, social media emerges as a swift and efficient channel for disseminating knowledge in dentistry; however, it is imperative to assess whether this information aligns with scientific evidence. This study aims to evaluate the quality of information found on public Instagram profiles in São Paulo State, Brazil, regarding the utilization of botulinum toxin (BTx) for bruxism treatment.

**Material and Methods:**

The data were categorized into three qualitative groups: information pertaining to bruxism diagnosis, treatment options for bruxism, and the application of BTx for bruxism. Following the selection of pertinent publications, 50 public profiles were included in the analysis. The publications were assessed utilizing the Global Quality Scale (GQS).

**Results:**

A total of 20,546 posts were tallied across the 50 profiles, with 230 relating to bruxism diagnosis, 166 discussing bruxism treatment options, and 78 mentioning the use of BTx for bruxism. Of these 78 posts addressing BTx for bruxism, 61% did not align with current scientific references, while 39% did. GQS analysis disclosed predominantly "poor quality" content (GQS = 2).

**Conclusions:**

It is concluded that the themes of bruxism and BTx are frequently broached on public Instagram profiles, yet the quality of the available information is generally subpar and often lacks scientific substantiation.

** Key words:**Botulinum Toxins, Type A; Bruxism; Online Social Networking; Sleep Bruxism.

## Introduction

Bruxism, characterized by repetitive masticatory muscle activity involving clenching or grinding of the teeth and/or mandibular bracing or thrusting, is a prevalent condition classified into sleep bruxism (SB) occurring during sleep, and awake bruxism (AB) manifesting when the individual is awake (1). Diagnosing bruxism presents challenges due to its dynamic nature, categorized into “possible,” “probable,” and “definite” levels based on various diagnostic criteria (1,2). Its prevalence ranges from 8% to 30% in the general population, with primary and secondary forms associated with diverse health conditions (3-5).

Despite its dental manifestations, bruxism is primarily regulated centrally, necessitating treatments aimed at managing its consequences rather than eradicating the condition itself (1,2). Current therapeutic approaches encompass occlusal splint therapy, cognitive-behavioral interventions, and lifestyle modifications to mitigate tooth wear, muscle fatigue, and orofacial pain associated with bruxism (9-11).

Botulinum toxin (BTx) injections have emerged as a potential intervention for bruxism, garnering interest among dental professionals for their purported efficacy in reducing muscle hyperactivity (12). The accessibility and perceived safety of BTx injections have fueled their popularity within the dental community, prompting increased discourse on social media platforms such as Instagram (13).

The internet, serving as a ubiquitous source of information, has become integral in disseminating medical knowledge, with patients and professionals alike turning to social networks for dental-related information. Instagram, renowned for its visual-centric approach and hashtag functionality, has emerged as a prominent platform for sharing dental insights and services (14-16).

Recent years have witnessed a surge in interest among dental professionals and patients regarding alternative treatments for bruxism, driven partly by the limitations of conventional therapies and the desire for more effective solutions. This has led to an exploration of novel interventions such as botulinum toxin injections, which have shown promise in alleviating bruxism symptoms by targeting the underlying muscle hyperactivity. However, despite anecdotal reports suggesting their efficacy, the scientific evidence supporting the use of botulinum toxin for bruxism remains limited and inconclusive, necessitating a comprehensive evaluation of its application and efficacy (15,16).

Moreover, the proliferation of information on social media platforms like Instagram has democratized access to healthcare knowledge, empowering individuals to seek information and advice beyond traditional healthcare channels. While this democratization of information has facilitated greater patient engagement and autonomy in healthcare decision-making, it has also raised concerns about the quality and accuracy of the information disseminated, particularly in the absence of professional oversight. Against this backdrop, examining the quality of information on Instagram regarding botulinum toxin for bruxism assumes critical importance, as it not only informs clinical practice but also shapes patient perceptions and expectations regarding treatment outcomes. By shedding light on the prevalence and reliability of such information, this study aims to provide valuable insights into the evolving landscape of bruxism management and the role of social media in shaping healthcare discourse.

This study aims to assess the quality of information available on public Instagram profiles in São Paulo State, Brazil, regarding the use of botulinum toxin (BTx) for bruxism. The data were categorized into three qualitative groups: information about bruxism diagnosis, bruxism treatment options, and the use of BTx for bruxism. After selecting relevant publications, 50 public profiles were included in the analysis. The publications were also evaluated using the Global Quality Scale (GQS), which is a standardized evaluation tool used to assess the quality and reliability of content, particularly in the context of online and digital information.

## Material and Methods

Data collection involved searching for profiles using specific hashtags related to bruxism and BTx. Profiles were included based on the number of followers, reach of posts, professional status, and the presence of relevant dental content. Profiles with less than 500 followers and fewer than 25 posts, as well as those unrelated to dentistry, were excluded. The hashtags used for profile selection included #tmdbotox, #botoxbruxism, #bruxism, #functionalbotox, #teethclenching, and #toothclenching. Posts were categorized into three groups: “information regarding the diagnosis of bruxism,” “information regarding bruxism treatment options,” and “information regarding the use of BTx to control bruxism.” The 50 selected public profiles contributed to the data analysis.

For posts discussing the use of BTx for bruxism, individual analysis was conducted based on seven relevant scientific references. Each post was categorized as either agreeing or disagreeing with these references (1,2,17,18-22).

The content of each post was scored for accuracy and reliability using a 5-point scale, which was used to assess the quality of posts according to the Global Quality Scale (GQS). The GQS assessed content quality, information coverage, and patient utility. The scale ranged from 1 (very poor quality) to 5 (excellent quality). Quantitative statistics were initially used to analyze the data, including the total number of publications, number of publications in each category, and overall average of publications per profile. Qualitative analysis was conducted through content analysis using the GQS and the agreement with current scientific evidence. It assigns a numerical score to content, often on a scale of 1 to 5, with higher scores indicating better quality. The GQS assesses various aspects of content, including accuracy, reliability, information coverage, and its utility to the intended audience. It is commonly employed in evaluating the educational quality of health-related content on the internet and other digital platforms.

All information analyses were conducted by two different researchers and cross-verified three times. In cases of significant discrepancies between the two assessors, the information was referred to a third assessor for final determination regarding the level of agreement with scientific evidence and the GQS score.

## Results

**Figure 1:**
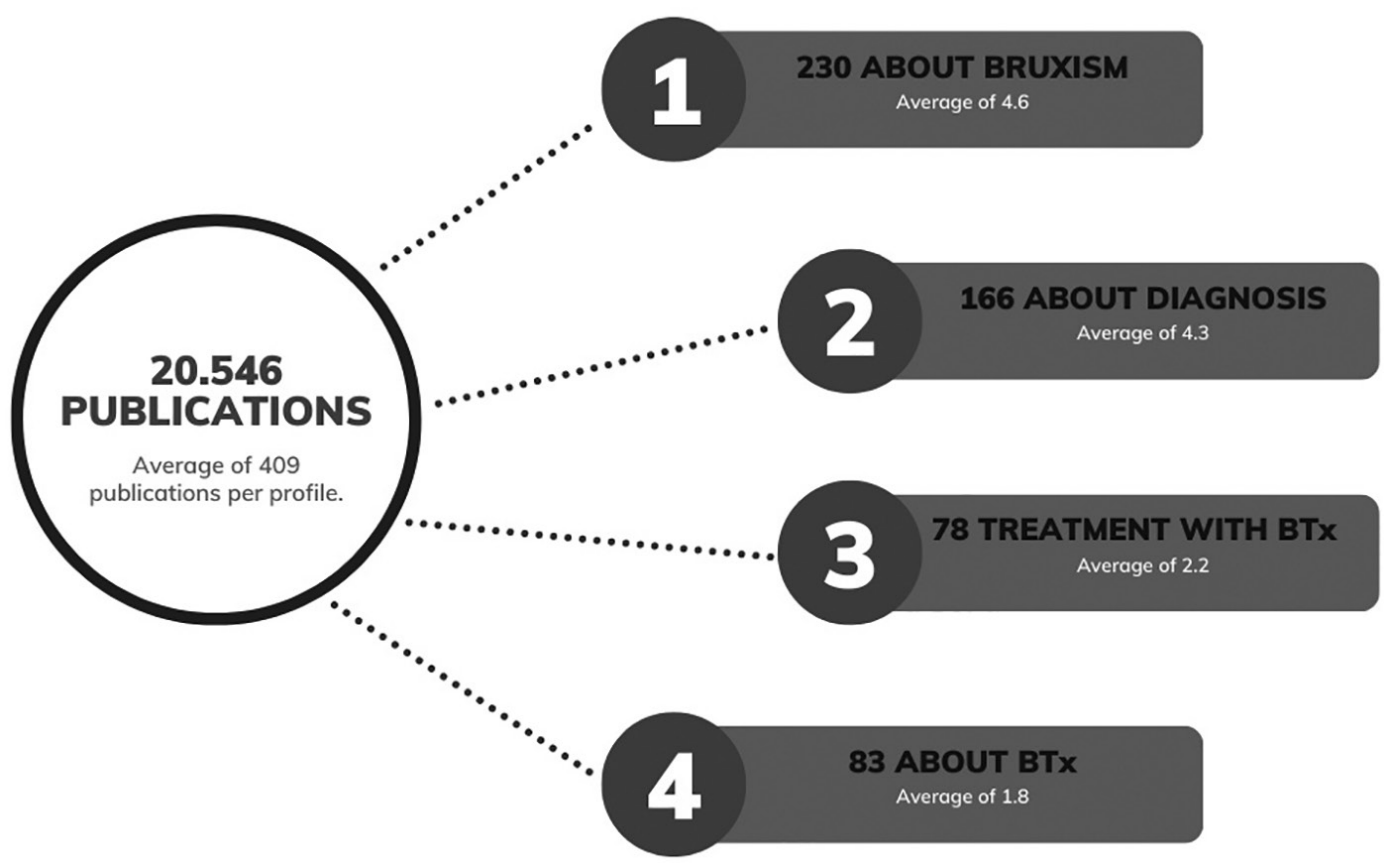
Total and average number of analyzed publications, average number of publications per profile, total and average number of publications on bruxism, publications on bruxism diagnosis, publications on the utilization of botulinum toxin for bruxism, and publications on botulinum toxin.

The analysis encompassed 50 public profiles from São Paulo State, Brazil, including 43 dentists (2 of whom specialized in TMD and Orofacial Pain), 3 physiotherapists, a dermatologist, and 3 professionals from other categories (biomedical and beauticians). The average number of publications per profile was 409, with an average of 4.6 publications related to “bruxism”. Among these publications, 230 were associated with bruxism diagnosis, 166 discussed bruxism treatment options, and 78 mentioned the use of BTx for bruxism (Fig. 1).

**Figure 2:**
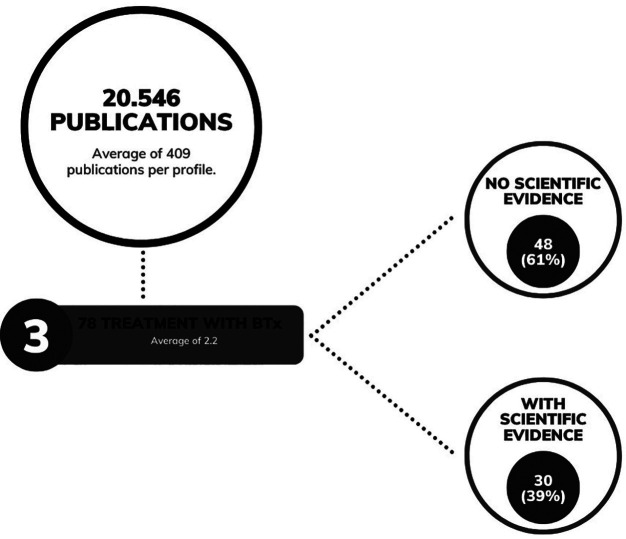
Total and average number of publications analyzed, along with publications regarding the utilization of botulinum toxin for bruxism. Total and percentage of publications related to the utilization of botulinum toxin for bruxism without scientific evidence and with scientific evidence, respectively.

Individual analysis of the posts related to the use of BTx for bruxism revealed that 48 of the 78 posts (61%) disagreed with current scientific references or presented clinical trial results lacking proven scientific effectiveness, while 30 posts (39%) aligned with scientific evidence (Fig. 2).

The analysis using the Global Quality Scale (GQS) exposed a prevailing presence of content categorized as “poor quality” (GQS=2), as indicated by evaluations of quality assessment, information coverage, and patient utility ( Table 1). Specifically, with regards to the quality assessment, 54% of the posts received a score of 1, while in terms of information coverage, 56% were assigned a score of 2. In the context of patient utility, 57% were given a score of 2.

## Discussion

Bruxism has central origin and is mediated by neurotransmitters such as dopamine (1-4). On the other hand, the action of BTx on bruxism is peripheral and does not act on its physiological parameters, being perceived only as a decrease in muscle strength. In 2014, Shim *et al*. (23) conducted a study involving individuals with sleep bruxism who received BTx injections and after injections maintained the same frequency, duration, and number of electromyographic events. In addition, despite the reduction in muscle strength, occlusal contacts continue to occur, favoring tooth wear (12,23,24). As far as the dentist is concerned, the consequences of bruxism are represented by wear and fractures of dental elements, failure of oral rehabilitations and orofacial pain (25-27), which means that the use of BTx does not dispense the use of an interocclusal splint to protect dental structures, supporting tissues and/or oral rehabilitations (27,28).

According to the results of the present study, most professionals who disclose about the application of BTx for bruxism through Instagram are not in line with the scientific evidence on the subject (61%) (1,2,18-22). Furthermore, they do not make a clear distinction between bruxism and TMD, treating both as same entities. Such errors may be associated with the training of the analyzed professionals, as only two (4%) were TMD and Orofacial Pain specialists. Another important point is the trivialization of the risks inherent to the procedure, found, for example, in posts that publicize the procedure for joint TMD and the application of BTx in this structure, demonstrating a lack of knowledge within the specialty, since bruxism is muscular and the toxin paralyzes the muscle (7,12). Regarding AB, which occurs when the patient is awake, no publications were found, as well as studies related to the use of BTx in awake bruxism (7,12), probably because it is a behavioral pattern, whose control must be directed through cognitive techniques (4,11).

According to the results of the GQS, more than half of the publications on the application of BTx for bruxism treatment have poor or generally poor educational quality (score < 3), in which the average found of this information for quality assessment, information coverage and patient utility was 2.04, 2.08 and 2, respectively. The GQS is a commonly used tool to evaluate the educational quality of health-related content on the internet ranging from 1 to 5 (1 = very poor quality and 5 = excellent quality). In another study that used the same method to evaluate professionals in the dentistry field regarding information about orthodontic treatments on the YouTube social network, they found that the average GQS result of the videos posted by professionals on the platform was between 2.6 and 3.229, like the averages found in this study. As in another study related to information about pulpotomy and pulp capping in YouTube videos, the mean GQS results for the educational quality were 1.63 and 1.70, respectively30, corroborating with the present study about the lack of accurate and reliable dental information on social media.

According to the literature, BTx can cause muscle atrophy because of fiber disuse and the possibility of osteopenia of the condyle due to the disbalance in the homeostasis of bone metabolism (20,31,32). It should be noted that the clinical impact of these effects has not been established. In addition, it is not possible to establish how long the patient will present bruxism, which makes the indefinite use of BTx unsupported, since its subsequent effects are to be dose-dependent, as well as its cost (33).

The application of BTx does not replace other established methods, such as the interocclusal splint, which prevent the destructive effects of bruxism on dental structures (23,24). Conservative treatments, without adverse effects, should still be the first choice to reduce myofascial pain and protect teeth (18,22). The current literature demonstrates that the use of BTx to control bruxism does not dispense the use of interocclusal splint and cognitive behavioral therapy, since tooth wear continues to occur, besides the possibility of undesirable biological consequences (18,22-24,31-33).

In a recent study, the quality of information provided in YouTube videos pertaining to the use of botulinum toxin injections for bruxism treatment was assessed. When the demographic data of the videos were compared with their utility scores, it was observed that the durations of videos rated as excellent and moderate were statistically significantly longer than those rated as poor. However, no statistically significant differences were identified in the utility scores concerning the number of views, likes, dislikes, and comments. A statistically significant correlation was noted between video demographic data and the source of upload. Videos uploaded by individual users exhibited longer durations and received higher numbers of likes, dislikes, and comments compared to other videos (16).

A similar study also assessed the quality of publicly shared on Instagram posts concerning masseter botox. The scores for information reliability in Instagram video posts under the hashtag #masseterbotox were notably low, with an average of 1.34 ± 1.28. There were no statistically significant differences observed in the number of views, information reliability, or GQS scores across various groups categorized by the source of the video posts. GQS scores were higher in videos presenting personal experiences compared to those focused on providing information and advertising. Consequently, the study’s authors concluded that clinicians should caution their patients regarding the unreliability of information on Instagram and direct them toward credible sources on social media. These findings closely parallel those identified in the present study (34).

The limitations of this study are associated with the number of evaluated posts, which could have been higher if private professional profiles were included; however, ethical concerns are involved in this matter. In addition to the limitations related to the Global Quality Scale (GQS), which is an instrument that encompasses only three variables for information analysis (quality assessment, information coverage, and patient utility), and there may be a subjective bias in the qualitative content analysis. However, this study found important and “red flags” results that much of the information on professional Instagram profiles about BTx for bruxism is not guided by scientific evidence. Hence, posts should emphasize the potential side effects, elevated costs, and limited effectiveness associated with this treatment. Posts endorsing BTx for bruxism treatment while dismissing the use of splints are grounded in clinical replication and do not align with current scientific evidence.

In summary, this study has shed light on the important and complex relationship between bruxism, its treatment with botulinum toxin (BTx), and the information disseminated through social media platforms like Instagram. It is evident that bruxism has central neurological origins and is governed by neurotransmitters, particularly dopamine. In contrast, BTx’s action in managing bruxism is peripheral, primarily reducing muscle strength. However, the application of BTx does not address the underlying physiological parameters of bruxism, leading to ongoing occlusal contacts and potential tooth wear. This study’s analysis of professional Instagram profiles reveals that a substantial portion of the information available is not aligned with current scientific evidence. Furthermore, this study highlights the risks inherent in BTx treatment, the need for a multidisciplinary approach to bruxism management, and the role of conservative treatments, such as interocclusal splints, in protecting dental structures. Overall, this research contributes to our understanding of the challenges and complexities in managing bruxism, particularly in the era of easily accessible and widely disseminated information on social media platforms.

## Conclusions

This cross-sectional observational analysis indicates that the topics of “bruxism” and “botulinum toxin” are relatively common on public Instagram profiles in São Paulo State, Brazil. However, the quality of the information available is generally poor and often unsupported by scientific evidence. The study suggests that much of the information provided to patients lacks a scientific basis and may lead to misguided clinical decision-making with limited efficacy.

## Figures and Tables

**Table 1:** Global Quality Scale (GQS) containing the frequency and average scores obtained for quality assessment, information coverage and patient utility.

Score	Quality assessment	Information coverage	Patient utility
1	54.43%	9.83%	25.9%
2	24.77%	56.29%	57.22%
3	14.82%	11.44%	10.04%
4	5.96%	22.42%	6.22%
5	0	0	0.6%
Mean	2.04	2.08	2

Source: Authors (2024).

## Data Availability

The datasets used and/or analyzed during the current study are available from the corresponding author.
